# Combined treatment with sacubitril/valsartan plus dapagliflozin in patients affected by heart failure with reduced ejection fraction

**DOI:** 10.3389/fcvm.2023.1097066

**Published:** 2023-03-22

**Authors:** Juan Jiang, Jie Gao, Xiuzhen Zhang, Yuanmin Li, Heqin Dang, Yanlin Liu, Wenwen Chen

**Affiliations:** ^1^ Department of Stomatology, The Second Affiliated Hospital of Shandong First Medical University, Tai'an, China; ^2^ Department of Pharmacy, The Second Affiliated Hospital of Shandong First Medical University, Tai'an, China; ^3^ Department of Cardiology, The Second Affiliated Hospital of Shandong First Medical University, Tai'an, China

**Keywords:** heart failure, sacubitril/valsartan, dapagliflozin, combined therapy, effect

## Abstract

**Background:**

Data about real-world effects of combined therapy with sacubitril/valsartan plus dapagliflozin in patients affected by heart failure (HF) with reduced ejection fraction (HFrEF) has not been widely reported. In this article, the benefits of dapagliflozin and sacubitril/valsartan respect to improvements of cardiac function in patients with HFrEF would be investigated.

**Methods:**

HF patients prescribed sacubitril/valsartan between January 2020 and January 2022 in a tertiary teaching hospital were selected using the Computerized Patient Record System. Patients were divided into two groups according to whether they were taking dapagliflozin. Clinical parameters at baseline and during follow-up were retrospectively collected and analyzed.

**Results:**

Total of 136 consecutive patients were recruited for this study. 72 patients treated with sacubitril/valsartan and dapagliflozin were assigned to Group A, and another 64 patients receiving sacubitril/valsartan monotherapy were assigned to Group B. After treatment with sacubitril/valsartan plus dapagliflozin for a median follow-up period of 189 days (IQR, 180–276), significant improvements of cardiac function were achieved in Group A. Median N-terminal pro-B-type natriuretic peptide (NT-proBNP) level was significantly decreased from 2585 pg/ml (1014–3702.5) to 1260.5 pg/ml (439.8–2214.3) (*P* < 0.001). Mean left ventricular ejection fraction (LVEF) improved from 34.7 ± 4.6% to 39.2 ± 7.5% (*P* < 0.001). Mean daily dose of loop diuretics decreased from 37.1 ± 17.3 mg/day to 25.9 ± 18.5 mg/day (*P* < 0.001). Regarding safety, both systolic blood pressure (*P* = 0.002) and diastolic blood pressure (*P* = 0.002) significantly decreased. For patients in Group B, significant improvements in mean LVEF (*P* < 0.001), decreases in mean daily dose of loop diuretics (*P* = 0.001) and reductions in diastolic blood pressure (*P* = 0.023) were observed. Strikingly, both median *Δ* NT-proBNP (*P* = 0.04) and median *Δ* LAD (*P* = 0.006) in Group A were more pronounced in comparison with those seen in Group B.

**Conclusions:**

The combined use of sacubitril/valsartan and dapagliflozin was associated with improved cardiac function in patents with HFrEF, and led to greater reductions in LAD and NT-proBNP levels compared to sacubitril/valsartan monotherapy. These findings suggest that the combination therapy may offer more potent cardiovascular benefits.

## Introduction

Heart failure (HF) is a global major public health problem, with frequent re-hospitalizations, high mortality rates, and poor quality of life (1–3). Neurohumoral antagonists including angiotensin-converting enzyme inhibitors (ACEIs), angiotensin-receptor blockers (ARBs), beta-blockers and mineralocorticoid receptor antagonists (MRAs) represent the cornerstones of modern HF therapy and have decreased the mortality and re-hospitalization rates of HF patients. However, clinical prognosis in patients with HF remains unsatisfactory (4). Therefore, novel drugs were required to improve the outcome of these patients.

As a first-in-class angiotensin receptor-neprilysin inhibitor (ARNI), sacubitril/valsartan brought new option for the treatment of HF (5, 6). The clinical trials and real-world studies have established its long-lasting efficacy in reducing the combined risk of death from cardiovascular causes or hospital admission for HF and improving several clinical, hemodynamic, and echocardiographic parameters (7–12). Additionally, dapagliflozin, sodium-glucose co-transporter-2 (SGLT-2) inhibitor, has been shown to reduce the composite of cardiovascular death or worsening HF in patients with heart failure with reduced ejection fraction (HFrEF) in the DAPA-HF (Dapagliflozin And Prevention of Adverse outcomes in Heart Failure) trial (13). Based on treatment benefits observed in the pivotal trials, both sacubitril/valsartan and dapagliflozin received a Class I indication in most international clinical practice guidelines (14, 15). Previous studies of sacubitril/valsartan or dapagliflozin had few patients taking both drugs simultaneously. As a result, it was hard to evaluate the potential incremental value of combined treatment with dapagliflozin plus sacubitril/valsartan compared to merely one drug. Based on the discovery of the lack of a treatment interaction between baseline sacubitril/valsartan use and randomized dapagliflozin therapy in DAPA-HF trial, Solomon et al. estimated indirectly that benefit of dapagliflozin plus sacubitril/valsartan would be additive (16). However, direct evidence was still required for clinical decision making. To bridge this research gap, the efficacy and safety of combined therapy with dapagliflozin and sacubitril/valsartan compared to sacubitril/valsartan monotherapy in patients with HFrEF would be investigated in this article.

## Materials and methods

### Study population

HF patients receiving therapy with sacubitril/valsartan between January 2020 and January 2022 in a tertiary teaching hospital were selected using the Computerized Patient Record System (CPRS). Patients were divided into two groups according to whether they were taking dapagliflozin at baseline. Patients were included if they were at least 18 years of age, had New York Heart Association (NYHA) functional classes II to IV, and LVEF ≤ 40% by echocardiography. The exclusion criteria were as follows: (1) patients lost to any follow-up, (2) sacubitril/valsartan and/or dapagliflozin discontinued at follow-up, (3) HF primarily resulting from right ventricular failure, pericardial disease, or congenital heart disease, and (4) patients with malignant tumors. This study was in accordance with the Declaration of Helsinki and approved by the ethics committee of the hospital.

### Dosage and follow-up interval

At baseline, first dose of sacubitril/valsartan was decided by physicians according to clinical conditions. If tolerated during follow-up, patients should be titrated to the maximum tolerated dose. While, the initial dose of dapagliflozin should be the target dose (10 mg daily) or the maximally tolerated dose. The follow-up interval for assessment of blood pressure, NYHA functional class, laboratory tests, and echocardiography could not be pre-specified in the present retrospective observational study. However, to evaluate the effectiveness of the medical therapy (the combination of sacubitril/valsartan and dapagliflozin vs. sacubitril/valsartan monotherapy), we investigated the above variables at the time closest to 6 months after the initiation of medical treatment. As a result, these variables were evaluated on a median of 189 (IQR 180-276) days after the initiation of treatment.

### Study parameters and data collection

Clinical characteristics, including age, gender, smoker, alcohol drinking, prior hospitalization for HF, duration of HF, HF aetiology, mean dose of sacubitril/valsartan, mean dose of dapagliflozin, comorbidities, and drugs, were recorded for every patients at baseline. Meanwhile, blood pressure, NYHA functional class, laboratory tests, echocardiography and loop diuretics dose in furosemide equivalents (furosemide 20 mg = torsemide 10 mg) aimed to evaluate efficacy and safety of therapeutic drugs should be collected at baseline and during follow-up.

### Statistical analyses

Quantitative variables were presented as mean ± standard deviation if normally distributed or as median and interquartile range if not normally distributed. Normality was checked by the Kolmogorov-Smirnov test. Categorical data were expressed as numbers and percentages. Continuous data were compared with the Student's *t*-test or the Mann-Whitney U test, and categorical data were compared with *χ*2 test. Statistical significance was set at a two-tailed *p*-value < 0.05. Statistics were performed using the SPSS Statistics 26.0 software (Chicago, IL, USA).

## Results

### General information and baseline characteristics

After applying both the inclusion and exclusion criteria, a total of 136 consecutive patients (mean age 68.9 ± 12.8 years, 69.1% male) were selected for this study. Baseline characteristics of enrolled patients are summarized in Table 1. For comparison between the two treatment strategies, 72 patients treated with sacubitril/valsartan combined with dapagliflozin were assigned to Group A, and another 64 patients receiving sacubitril/valsartan without dapagliflozin were assigned to Group B.

**Table 1:** Baseline characteristics of enrolled patients.

Variable	Total (*n* = 136)	Group A (*n* = 72)	Group B (*n* = 64)	*P*
**Demographics**
Mean age, years	68.9 ± 12.8	67.6 ± 12.6	70.3 ± 12.9	0.235
Male, *n* (%)	94 (69.1)	49 (68.1)	45 (70.3)	0.776
Active smoker, *n* (%)	56 (41.2)	26 (36.1)	30 (46.9)	0.203
Alcohol drinking, *n* (%)	48 (35.3)	24 (33.3)	24 (37.5)	0.612
Prior hospitalization for HF, *n* (%)	92 (67.6)	40 (55.6)	52 (81.3)	0.001
Duration of HF, days	418.3 ± 95.6	410.8 ± 88.3	424.1 ± 101.4	0.510
HF aetiology, *n* (%)				1.000
Ischaemic	119 (87.5)	63 (87.5)	56 (87.5)	
Non-ischaemic	17 (12.5)	9 (12.5)	8 (12.5)	
Mean dose of sacubitril/valsartan, mg/day	102.6 ± 65.3	107.1 ± 67.7	97.7 ± 62.6	0.403
Mean dose of dapagliflozin, mg/day	10 ± 0	10 ± 0	10 ± 0	NA
**Comorbidities, *n* (%)**
Ischaemic heart disease	119 (87.5)	63 (87.5)	56 (87.5)	1.000
Atrial fibrillation	38 (27.9)	13 (18.1)	25 (39.1)	0.006
Hypertension	88 (64.7)	49 (68.1)	39 (60.9)	0.386
Diabetes	72 (52.9)	64 (88.9)	8 (12.5)	<0.001
Stroke	14 (10.3)	7 (9.7)	7 (10.9)	0.816
Median number of comorbidities	3 (2–3)	3 (2–3)	2 (1–3)	<0.001
**Drugs, *n* (%)**
Beta-blockers	88 (64.7)	55 (76.4)	33 (51.6)	0.002
Aldosterone antagonist	102 (75)	48 (66.7)	54 (84.4)	0.017
Loop diuretics	104 (76.5)	49 (68.1)	55 (85.9)	0.014
Digoxin	14 (10.3)	5 (6.9)	9 (14.1)	0.173
Anticoagulants	36 (26.5)	15 (20.8)	21 (32.8)	0.114
Statins	112 (82.4)	59 (81.9)	53 (82.8)	0.895
Aspirin	76 (55.9)	47 (65.3)	29 (45.3)	0.019
P2Y12 antagonists	77 (56.6)	45 (62.5)	32 (50.0)	0.142
Metformin	27 (19.9)	26 (36.1)	1 (1.6)	<0.001
Amiodarone	7 (5.1)	4 (5.6)	3 (4.7)	0.819
Median number of drugs	4 (4–5)	5 (4–6)	4 (3–5)	<0.001
**Blood pressure**
Mean SBP, mmHg	135.9 ± 23.7	136.7 ± 23.3	135.1 ± 24.4	0.704
Mean DBP, mmHg	80.8 ± 14.9	81.0 ± 13.2	80.4 ± 16.7	0.810
**Laboratory values**
Mean potassium, mmol/L	4.0 ± 0.5	4.0 ± 0.5	4.0 ± 0.6	0.809
Median serum creatinine, mg/dl	0.9 (0.7–1.1)	0.8 (0.7–1.0)	1.0 (0.8–1.2)	0.001
Median BUN, mmol/L	7.3 (5.6–9.1)	7.0 (5.6–8.6)	7.6 (5.4–10.6)	0.275
Median NT-proBNP, pg/mL	2,585 (841.25–4105.85)	2,585 (1014–3702.5)	2720.5 (841.25–4322)	0.965
NYHA classification, n (%)				0.001
Class I/II	47 (34.6)	34 (47.2)	13 (20.3)	
Class III/IV	89 (65.4)	38 (52.8)	51 (79.7)	
**Echocardiography data**
Mean LVEF, %	34.8 ± 4.8	34.7 ± 4.6	34.9 ± 5.1	0.774
Median LVEF, %	36 (32–39)	35 (32–38)	37 (31–39)	0.621
Median LVEDD, mm	58 (53–61.75)	57 (53–61)	58 (52–64)	0.519
Median LAD, mm	46 (42–51)	46 (43–50)	45 (41–51)	0.088
Median RVEDD, mm	24 (22–24)	23 (22–24)	24 (21–25)	0.264
Mean loop diuretics dose, mg/day	39.0 ± 17.9	37.1 ± 17.3	40.7 ± 18.4	0.311

HF, heart failure; SBP, systolic blood pressure; DBP, diastolic blood pressure; BUN, blood urea nitrogen; NT-proBNP, N-terminal pro-B-type natriuretic peptide; NYHA, New York Heart Association; LVEF, left ventricular ejection fraction; LVEDD, left ventricular end-diastolic diameter; LAD, left atrium diameter; RVEDD, right ventricular end diastolic dimension; NA, not available.

As shown in Table 1, compared with patients in Group B, patients receiving combination treatment (Group A) were less likely to have a prior hospitalization for HF, had better baseline NYHA functional class, higher number of comorbidities and higher number of drugs, were less likely to have a history of atrial fibrillation and more likely to have a history of diabetes, had lower serum creatinine, and were more often treated with beta-blockers, aspirin, and metformin and less likely to have received aldosterone antagonist and loop diuretics. Other variables, including age, gender, active smoker, alcohol drinking, duration of HF, HF aetiology, maintained dose of sacubitril/valsartan, history of ischaemic heart disease, hypertension, or stroke, use of digoxin, anticoagulants, statins, P2Y12 antagonists, and amiodarone, systolic blood pressure (SBP), diastolic blood pressure (DBP), laboratory tests, echocardiography data, and loop diuretics dose were similar between Group A and Group B.

### Intra-group comparisons of clinical parameters from baseline to follow-up

**Figure 1:**
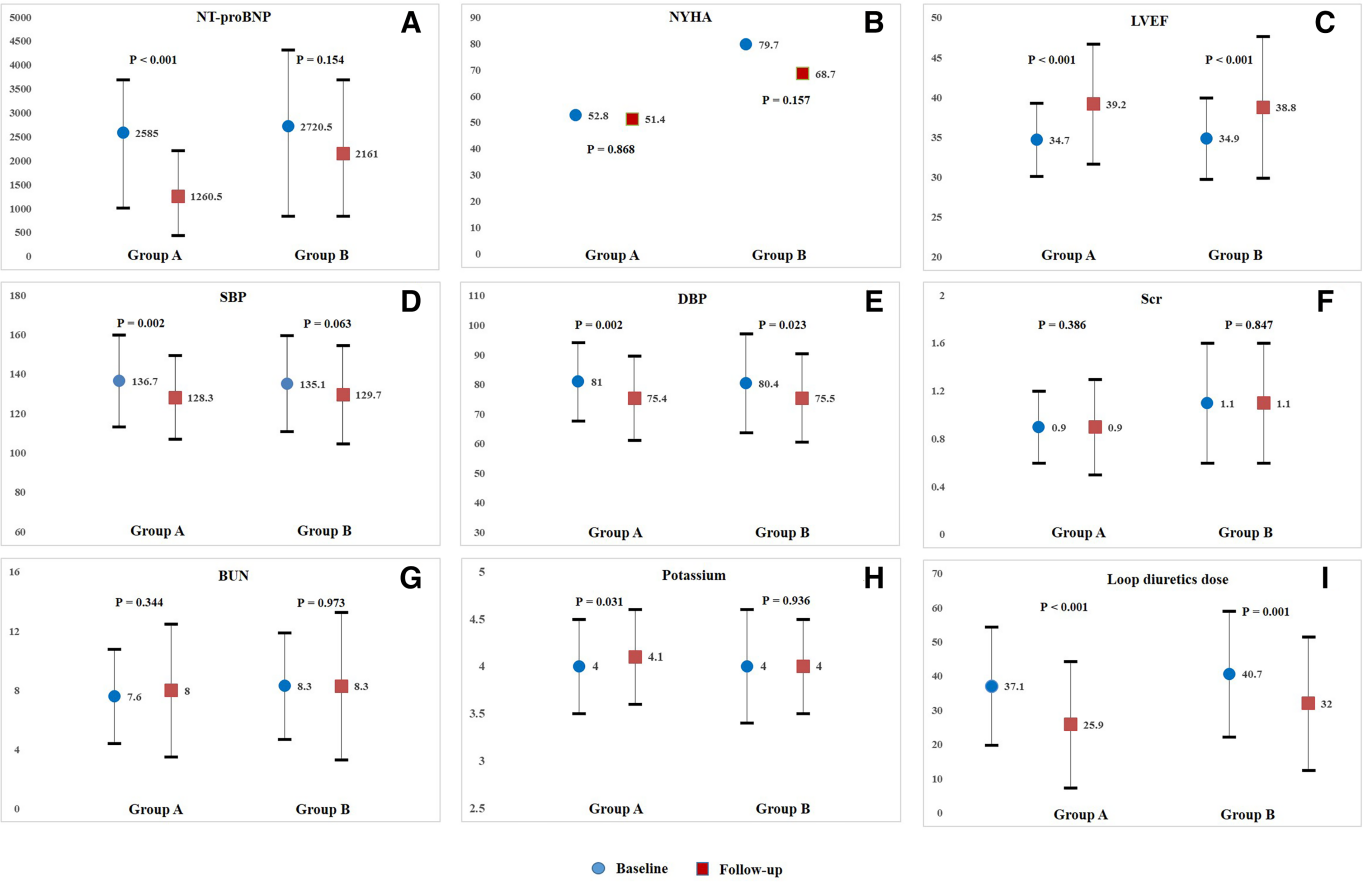
Intra-group comparisons of NT-proBNP(**A**), NYHA class (**B**), LVEF(**C**), SBP(**D**), DBP(**E**), Scr(**F**), BUN(**G**), potassium(**H**), and loop diuretics dose(**I**), from baseline to follow-up in Group A or Group B. NT-proBNP, N-terminal pro-B-type natriuretic peptide; NYHA, New York Heart Association; LVEF, left ventricular ejection fraction; SBP, systolic blood pressure; DBP, diastolic blood pressure; Scr, serum creatinine; BUN, blood urea nitrogen.

In Group A, after treatment with sacubitril/valsartan plus dapagliflozin for a median follow-up period of 189 days (IQR, 180–276), median N-terminal pro-B-type natriuretic peptide (NT-proBNP) level was significantly decreased from 2585 pg/ml (1014–3702.5) to 1260.5 pg/ml (439.8–2214.3) (*P* < 0.001) (Figure 1A). The proportion of patients in NYHA Class III/IV decreased slightly from 52.8% to 51.4% (*P* = 0.868) (Figure 1B). Moreover, noticeable improvements in a series of echocardiographic parameters were also observed during follow-up. Mean left ventricular ejection fraction (LVEF) improved from 34.7 ± 4.6% to 39.2 ± 7.5% (*P* < 0.001) (Figure 1C). Median left ventricular end-diastolic diameter (LVEDD) decreased from 57 mm (IQR, 53–61) to 56 mm (IQR, 50.3–60) (*P* = 0.042). Median left atrium diameter (LAD) decreased from 46 mm (IQR, 43–50) to 44.5 mm (IQR, 40–48) (*P* = 0.003). The mean daily dose of loop diuretics in furosemide equivalents decreased from 37.1 ± 17.3 mg/day to 25.9 ± 18.5 mg/day (*P* < 0.001) (Figure 1I). Regarding safety, both SBP (from 136.7 ± 23.3 mmHg to 128.3 ± 21.2 mmHg, *P* = 0.002) and DBP (from 81 ± 13.2 mmHg to 75.4 ± 14.3 mmHg, *P* = 0.002) significantly reduced after combined treatment (Figures 1D,E). Median serum creatinine level (Figure 1F) and median blood urea nitrogen (BUN) level (Figure 1G) did not change obviously during follow-up, but mean potassium (Figure 1H) decreased distinctly. Additionally, hypotension (SBP < 100 mmHg) occurred only in 1 patients during the treatment period.

For patients in Group B, only mean LVEF improved significantly from 34.9 ± 5.1% to 38.8 ± 8.9% (*P* < 0.001) (Figure 1C), but no obvious improvements in other echocardiographic parameters were observed at follow-up. Additionally, mean daily dose of loop diuretics also decreased significantly from 40.7 ± 18.4 mg/day to 32.0 ± 19.5 mg/day (*P* = 0.001) (Figure 1I). Both median NT-proBNP level (*P* = 0.154) and the proportion of patients in NYHA Class III/IV decreased slightly (*P* = 0.157) (Figures 1A,B). Regarding safety, only DBP significantly reduced from 80.4 ± 16.7 mmHg to 75.5 ± 14.9 mmHg (*P* = 0.023) (Figure 1E). No obvious changes were observed in SBP (Figure 1D), median serum creatinine level (Figure 1F), median BUN level (Figure 1G), and mean potassium level (Figure 1H) from baseline to follow-up. Over the entire treatment period, hypotension occurred in 5 patients, and no drug withdrawal occurred in those patients. Intra-group comparisons of clinical parameters from baseline to follow-up in Group A or Group B were illustrated in Figure 1 and Supplementary Table S1.

### Comparisons of changes in clinical parameters from baseline to follow-up between group A and group B

Table 2 illustrates comparative analysis of changes in clinical parameters from baseline to follow-up between Group A and Group B. Strikingly, both median *Δ* NT-proBNP (*P* = 0.04) and median *Δ* LAD (*P* = 0.006) in Group A were more pronounced in comparison with those seen in Group B. Changes in other clinical parameters in Group A were also obvious compared with Group B, but there were no statistically significant differences.

**Table 2:** Comparisons of changes in clinical parameters from baseline to follow-up between group A and group B.

Variable	Group A (*n* = 72)	Group B (*n* = 64)	*P*
**Blood pressure**
Mean *Δ* SBP, mmHg	−8.4 ± 22.7	−5.4 ± 22.8	0.439
Mean *Δ* DBP, mmHg	−5.6 ± 14.6	−4.7 ± 16.3	0.738
**Laboratory values**
Mean *Δ* potassium, mmol/L	0.2 ± 0.6	0 ± 0.7	0.144
Median *Δ* serum creatinine, mg/dl	0 ± 0.3	0 ± 0.5	0.838
Median *Δ* BUN, mmol/L	−0.1 (-1.7-2.1)	−0.2 (-2.2 1.6)	0.464
Median *Δ* NT-proBNP, pg/mL	−972 (-2195.3-30)	−212.2 (-1567.9-764.4)	0.04^a^
**Echocardiography data**
Mean *Δ* LVEF, %	4.5 ± 6.0	3.9 ± 8.0	0.622
Median *Δ* LVEDD, mm	−1 (-3.8-1.8)	0 (-3.8-3)	0.323
Median *Δ* LAD, mm	−1 (-5-1)	0 (-2.8-4)	0.006^a^
Median *Δ* RVEDD, mm	−1 (-1-1)	0 (-3-2)	0.795
Mean *Δ* loop diuretics dose, mg/day	11.2 ± 16.0	8.7 ± 17.5	0.452

^a^ After adjusting the prevalence of diabetes and prior hospitalization for HF, median *Δ* NT-proBNP and median *Δ* LAD were also significantly different between Group A and Group B (adjusted *P* = 0.011 for median *Δ* NT-proBNP and adjusted *P* = 0.008 for median *Δ* LAD).

SBP, systolic blood pressure; DBP, diastolic blood pressure; Scr, serum creatinine; BUN, blood urea nitrogen; NT-proBNP, N-terminal pro-B-type natriuretic peptide; NYHA, New York Heart Association; LVEF, left ventricular ejection fraction; LVEDD, left ventricular end-diastolic diameter; LAD, left atrium diameter; RVEDD, right ventricular end diastolic dimension.

## Discussion

In this article, our data supported the evidence that combined therapy with sacubitril/valsartan plus dapagliflozin could effectively improve cardiac function and was well tolerated in Chinese patients with HFrEF. Furthermore, we showed that combined therapy with sacubitril/valsartan plus dapagliflozin led to greater reductions in LAD and NT-proBNP levels compared to sacubitril/valsartan monotherapy.

As novelty in HF therapy, sacubitril/valsartan and dapagliflozin, which reduced cardiovascular mortality and morbidity in randomized controlled trials, had emerged as evidence-based therapies for HF (7, 13, 17–20). 2021 ESC guideline on HF gave a Class I recommendation for the use of sacubitril/valsartan and dapagliflozin in HFrEF patients, and required that two drugs should be initiated simultaneously and up-titrated rapidly (21). Worth noting, patients in real-world clinical practice had many comorbidities, which might influenced therapeutic regimen (11, 12). In the present study, patients received combined treatment were more likely to have a history of diabetes mellitus compared with those taking sacubitril/valsartan monotherapy. Consistently, in a recently published study, the proportion of patients who were comorbid with diabetes mellitus in the sacubitril/valsartan plus dapagliflozin group was significantly higher than that in sacubitril/valsartan group (74.1% vs. 51.9%, *P* = 0.001) (22). This gives us a hint that patients with HFrEF and concomitant diabetes mellitus are more likely to be treated with sacubitril/valsartan plus dapagliflozin. Additionally, in February 2021, dapagliflozin was approved to treat HF in China. Therefore, the use of dapagliflozin for managing HF had been limited to diabetic patients before approval, which might result in difference in the prevalence of diabetes mellitus between Group A and Group B.

Another thing to be noted is that the mean maximum tolerated dose of sacubitril/valsartan achieved in Group A (107.1 ± 67.7 mg) or Group B (97.7 ± 62.6 mg) is lower than that achieved in PARADIGM-HF trial (7). Indeed, low dose of sacubitril/valsartan is very common in real-world clinical setting due to several factors (symptomatic hypotension, hyperkalemia, renal dysfunction and worsening heart failure), which is a clear difference from landmark trial (23–27). In a prospective observational cohort study, even under therapy with low-dose sacubitril/valsartan (135.9 ± 75.5 mg), significant decrease in NT-proBNP concentration (from 2,495 pg/ml to 943 pg/ml, *P* < 0.001) and prominent increase in the LVEF (from 35.6% ± 10% to 47.% ± 14.2%, *P* < 0.001) were observed (28). Another real-world study also confirmed that low-dose sacubitril/valsartan (122.5 ± 55.2 mg) significantly reduced NT-proBNP (from 3,003 pg/mL to 2,039 pg/mL, *P* = 0.010), improved NYHA classification (*P* < 0.001), and induced beneficial cardiac reverse remodeling (LVEF increased from 31 ± 6% to 38 ± 10%, *P* < 0.001) (11). Additionally, sacubitril/valsartan showed well tolerability, and fewer patients discontinued sacubitril/valsartan due to hypotension or abnormal laboratory values (11, 28). Consistently, patients receiving the low dosage of sacubitril/valsartan monotherapy (Group B) in this article also achieved prominent increase in LVEF (from 34.9 ± 5.1% to 38.8 ± 8.9%, *P* < 0.001). However, improvement in the NYHA class (*P* = 0.157) and reduction in NT-proBNP (*P* = 0.154) concentration was not significant, which could be explained by that daily dose of sacubitril/valsartan in this study was lower than that in the previous real-world studies (11, 26, 28). Fortunately, in patients treated with sacubitril/valsartan and dapagliflozin (Group A), significant decrease in median NT-proBNP level (from 2,585 pg/ml to 1260.5 pg/ml, *P* < 0.001), as well as pronounced improvements in left cardiac remodeling measurements including LVEF (*P* < 0.001), LAD (*P* = 0.003) and LVEDD (*P* = 0.042) were observed. Of note, daily dose of sacubitril/valsartan in Group A was as low as that in Group B.

Furthermore, both median *Δ* NT-proBNP (unadjusted *P* = 0.04 and adjusted *P* = 0.011) and median *Δ* LAD (unadjusted *P* = 0.006 and adjusted *P* = 0.008) in Group A were more remarkable in comparison with those seen in Group B. This discovery revealed potential incremental value of treatment with both sacubitril/valsartan and dapagliflozin. In a retrospective observational study, long-term cardiac mortality rates in the sacubitril/valsartan plus dapagliflozin group (7.4%) were significantly lower than that in the sacubitril/valsartan monotherapy group (19.5%) (*P* = 0.01) (22). In another study conducted in diabetic patients with HFrEF, combination of ARNI and SGLT2 inhibitors could improve the clinical course of HFrEF in patients compared to ARNI monotherapy (29). Patients treated with combination of ARNI and SGLT2 inhibitors exhibited a lower risk of hospitalization for HF or cardiovascular mortality (*P* = 0.04) compared to those treated with ARNI only. Additionally, patients treated with combination of ARNI and SGLT2 inhibitors tended to show higher LVEF than those treated with ARNI only throughout the follow-up period. However, these differences were not statistically significant, which might attenuate incremental value of combined treatment with ARNI plus SGLT2 inhibitors on echocardiographic parameters compared to merely ARNI (29). Notably, in a study reported by Hwang et al., HF patients treated with SGLT2 inhibitors showed a significant decrease in LVEDD (*P* < 0.001) and improvement in LVEF (*P* < 0.001) (30). Therefore, further studies are required to investigate the effective mechanism of action of SGLT2 inhibitors when it is added to ARNI treatment regimens. In a subgroup analysis of the DAPA-HF trial, Solomon et al. discovered indirectly that the use of sacubitril/valsartan and dapagliflozin together could further lower morbidity and mortality in patients with HFrEF without compromising safety (16). The results in these studies provided evidence that the clinical benefits of treatment with both sacubitril/valsartan and dapagliflozin might be greater than sacubitril/valsartan monotherapy.

It is worth noting that differences in the prevalence of diabetes and incidence of prior hospitalization for HF between Group A and Group B at baseline in the study might impact incremental value of combined treatment with dapagliflozin plus sacubitril/valsartan compared to merely sacubitril/valsartan. As a common co-morbidity in patients suffering from HF, diabetes mellitus is a well-established risk factor for worse outcome in HF, and is associated with increased hospitalization and mortality rates in chronic HF (31). Diabetes can contribute to HF development and progression in multiple ways including metabolic and functional alterations, hyperglycemia-induced structural abnormalities, microvascular dysfunction, cardiac autonomic neuropathy, and neurohormonal abnormalities (32–34). Compared with nondiabetics, diabetics seem to have higher BNP levels (35) and depressed systolic function (36). Therefore, conclusion that the clinical benefits of treatment with both sacubitril/valsartan and dapagliflozin might be greater than sacubitril/valsartan monotherapy should be treated with caution due to differences in prevalence of diabetes between Group A and Group B at baseline. Hospitalization for HF represents a destabilizing event in the clinical trajectory of patients with HF (37). It should be stated that the incidence of prior hospitalization for HF was significantly higher in Group B compared to Group A at baseline in the study. This difference might suggest that patients in Group B had more advanced or prolonged HF, potentially attenuating the benefits of medical therapy. However, as another factor associated with poor outcome in HF (38), duration of HF was similar between Group A and Group B in the present study. This gave us a hint that patients in each group might have similar progression of HF. In the future, large-sample and multicenter studies are required to explore the effect of incidence of prior hospitalization for HF on the benefits of medical therapy.

Several limitations in the retrospective study should be mentioned. First, overall number of patients recruited in the current study was relatively small. Second, echocardiography data was evaluated by 2D-echocardiographic assessment in our study, which was not as accurate as 3D-echocardiography. Third, the maintenance dosage of sacubitril/valsartan was relatively low. Therefore, the optimal dosage of sacubitril/valsartan should be explored in the future. Scheduled drug-escalation programs which might be helpful to achieve higher daily dose of sacubitril/valsartan were required to establish for patients with HFrEF. Fourth, indicators of congestion, including central venous pressure and pulmonary capillary wedge pressure, were not measured in patients. Therefore, clinical data on the benefit of congestion could not be provided in the present study.

## Conclusion

In patients with HFrEF, treatment with the combination of sacubitril/valsartan and dapagliflozin was associated with improved cardiac function, and resulted in greater reductions in LAD and NT-proBNP levels compared to sacubitril/valsartan monotherapy. These data would expand the combined use of sacubitril/valsartan and dapagliflozin as a daily routine in clinical practice if supported by more high-quality, large-sample, multicenter studies in the future.

## Data Availability

The raw data supporting the conclusions of this article will be made available by the authors, without undue reservation.
