# Supplementary material for: Combined treatment with sacubitril/valsartan plus dapagliflozin in patients affected by heart failure with reduced ejection fraction
Source: Front Cardiovasc Med. 2023 Mar 22;10:1097066. doi: 10.3389/fcvm.2023.1097066 (PMC10073490; doi:10.3389/fcvm.2023.1097066)
Supplement: Supplementary file 1 [file Table1.docx]

Supplemental Table 1 Intra-group comparisons of clinical parameters from baseline to follow-up in Group A or Group B.

| Variable | Group A (n=72) | | P | Group B (n=64) | | P |
| --- | --- | --- | --- | --- | --- | --- |
|  | Baseline | Follow-up |  | Baseline | Follow-up |  |
| Blood pressure |  |  |  |  |  |  |
| Mean SBP, mmHg | 136.7 ± 23.3 | 128.3 ± 21.2 | 0.002 | 135.1 ± 24.4 | 129.7 ± 24.9 | 0.063 |
| Mean DBP, mmHg | 81 ± 13.2 | 75.4 ± 14.3 | 0.002 | 80.4 ± 16.7 | 75.5 ± 14.9 | 0.023 |
| Laboratory values |  |  |  |  |  |  |
| Mean potassium, mmol/L | 4.0 ± 0.5 | 4.1 ± 0.5 | 0.031 | 4 ± 0.6 | 4 ± 0.5 | 0.936 |
| Median serum creatinine, mg/dl | 0.9 ± 0.3 | 0.9 ± 0.4 | 0.386 | 1.1 ± 0.5 | 1.1 ± 0.5 | 0.847 |
| Median BUN, mmol/L | 7.6 ± 3.2 | 8.0 ± 4.5 | 0.344 | 8.3 ± 3.6 | 8.3 ± 5.0 | 0.973 |
| Median NT-proBNP, pg/mL | 2585 (1014-3702.5) | 1260.5 (439.8-2214.3) | < 0.001 | 2720.5 (841.3-4322) | 2161 (841.3-3700) | 0.154 |
| NYHA classification, n (%) |  |  | 0.868 |  |  | 0.157 |
| Class I/II | 34 (47.2) | 35 (48.6) |  | 13 (20.3) | 20 (31.3) |  |
| Class III/IV | 38 (52.8) | 37 (51.4) |  | 51 (79.7) | 44 (68.7) |  |
| Echocardiography data |  |  |  |  |  |  |
| Mean LVEF, % | 34.7 ± 4.6 | 39.2 ± 7.5 | < 0.001 | 34.9 ± 5.1 | 38.8 ± 8.9 | < 0.001 |
| Median LVEDD, mm | 57 (53-61) | 56 (50.3-60) | 0.042 | 58 (52-64) | 57 (51-66) | 0.639 |
| Median LAD, mm | 46 (43-50) | 44.5 (40-48) | 0.003 | 45 (41-51) | 47 (42-54) | 0.284 |
| Median RVEDD, mm | 23 (22-24) | 23 (21-23) | 0.079 | 24 (21-25) | 24 (20-26) | 0.224 |
| Mean loop diuretics dose, mg/day | 37.1 ± 17.3 | 25.9 ± 18.5 | < 0.001 | 40.7 ± 18.4 | 32 ± 19.5 | 0.001 |

SBP, systolic blood pressure; DBP, diastolic blood pressure; BUN, blood urea nitrogen; NT-proBNP, N-terminal pro-B-type natriuretic peptide; NYHA, New York Heart Association; LVEF, left ventricular ejection fraction; LVEDD, left ventricular end-diastolic diameter; LAD, left atrium diameter; RVEDD, right ventricular end diastolic dimension.
